# Long‐term imoxin treatment does not attenuate disease severity in dystrophic diaphragm of male mice

**DOI:** 10.14814/phy2.71115

**Published:** 2026-09-21

**Authors:** Ji Heun Lee, Morgan E. Vorwald, Melissa Roths, Joshua T. Selsby, Rudy J. Valentine

**Affiliations:** ^1^ Department of Kinesiology Iowa State University Ames Iowa USA; ^2^ Department of Animal Science Iowa State University Ames Iowa USA; ^3^ Department of Physical Therapy & Kinesiology University of Massachusetts Lowell Lowell Massachusetts USA

**Keywords:** DMD, Duchenne muscular dystrophy, dystrophin, ER stress, protein kinase R

## Abstract

Duchenne muscular dystrophy (DMD) is characterized by chronic skeletal muscle injury and degeneration. We discovered activation of protein kinase R (PKR; EIF2AK2), a regulator of the integrated stress response, in dystrophic muscle; however, its role in DMD remains unknown. We hypothesized that the PKR inhibitor imoxin (IMX) would improve muscle force and attenuate fibrosis, inflammatory signaling, and ER stress in diaphragms from mdx mice. C57 and mdx mice were treated with vehicle or IMX for ~24 wks (0.5 mg/kg, subcutaneous, 5 times/wk). Specific force was decreased (*p* < 0.001), and dynamic passive force was increased (*p* = 0.001) by disease, but there were no IMX effects. Disease‐increased fibrotic area was decreased by IMX (*p* = 0.038); however, IMX failed to reduce fibronectin, collagen, or TGF‐β1 signaling proteins. PKR and phosphorylated (p)PKR (thr446) were increased in mdx (*p* < 0.001) and maintained despite IMX treatment. Similarly, PKR regulators (total PACT, pPACT (ser18), TRBP, and PP1α), and PKR substrates (total eIF2α and peIF2α (ser51)) were also increased with disease (*p* < 0.001), but unaffected by IMX. Likewise, IMX did not attenuate disease‐mediated elevations in inflammatory signaling and ER stress markers. Overall, the dose of IMX was insufficient to attenuate PKR activation and failed to improve muscle function or prevent pathology in dystrophic skeletal muscle.

## 1 INTRODUCTION

Duchenne muscular dystrophy (DMD) is the most common, fatal X‐linked disease (Mah et al., 2014), and is caused by a dystrophin protein deficiency (Duan et al., 2021). Dystrophin deficiency results in a host of cellular pathologies that include increased inflammatory signaling (Chen et al., 2005; Monici et al., 2003) and ER stress (Hulmi et al., 2016; Krishna, Spaulding, et al., 2023; Moorwood & Barton, 2014; Pauly et al., 2017) that contribute to disease progression. DMD is characterized by progressive muscle injury, which ultimately leads to muscle weakness and impaired function (Birnkrant et al., 2018), reliance on ventilatory support (McKim et al., 2013), and culminates in patient death due to cardio‐pulmonary dysfunction (Landfeldt et al., 2020; Pennati et al., 2020).

Protein kinase R (PKR; EIF2AK2) is a key part of the integrated stress response, an adaptive signaling pathway intended to maintain cellular homeostasis, and has been implicated as a contributor to a variety of disease states, including neurodegenerative conditions (Hugon et al., 2017; Martinez et al., 2021), cancer (Marchal et al., 2014), and metabolic diseases (Carvalho et al., 2013; Nakamura et al., 2010). PKR activation leads to inhibition of global protein synthesis through phosphorylation of eukaryotic initiation factor 2 alpha subunit (eIF2α) at serine 51, thus blunting translation initiation (Costa‐Mattioli & Walter, 2020; Pakos‐Zebrucka et al., 2016). Canonically, PKR is activated by double‐stranded RNA (Meurs et al., 1990), but can also be activated by inflammatory signaling, oxidative stress, and ER stress (Gal‐Ben‐Ari et al., 2018). Further, once activated, PKR can promote inflammatory signaling through NFκB, MAPK, or NLRP3 inflammasome pathways (Bonnet et al., 2000; Chukwurah et al., 2021; Goh et al., 2000; Lu et al., 2012). Moreover, PKR activation has been implicated in ER stress and muscle proteolysis, as PKR inhibition reduced markers of ER stress (Eo & Valentine, 2021; Eo & Valentine, 2022) and attenuated lipopolysaccharide (LPS)‐induced expression of the E3 ubiquitin ligases, MuRF1 and MAFbx (Valentine et al., 2018). Notably, ER stress has been reported in dystrophic muscle (Hulmi et al., 2016; Krishna, Spaulding, et al., 2023; Moorwood & Barton, 2014; Pauly et al., 2017) and appears to contribute to disease progression (Moorwood & Barton, 2014; Pauly et al., 2017); however, the causal factor triggering ER stress in dystrophic muscle is not fully understood.

In dystrophic muscle, we previously discovered that PKR expression and activation were elevated (Krishna, Echevarria, et al., 2023; Krishna, Spaulding, et al., 2023), raising the possibility that PKR activity contributes to dystrophic pathology via promotion of inflammatory signaling and ER stress. However, the role of PKR and its impact on ER stress and inflammatory signaling in dystrophic muscle remains unknown. In this study, we applied the small‐molecule PKR‐inhibitor imoxin (IMX), which has been shown to alleviate ER stress in muscle cells (Eo & Valentine, 2021; Eo & Valentine, 2022) and LPS‐induced inflammation and atrophy genes in rodent muscle (Valentine et al., 2018). The purpose of this study was to investigate the effect of PKR inhibition on disease progression in mdx mice. We hypothesized that long‐term PKR inhibition would preserve muscle function and attenuate ER stress and inflammatory signaling in dystrophin‐deficient muscle.

## 2 MATERIALS AND METHODS

### 2.1 Animals and experimental design

All animal work was approved by the IACUC at Iowa State University (ISU; IACUC‐23‐110) and the University of Florida (202108822). Male mice were produced from our C57BL/10ScSnJ (C57) and C57BL/10ScSn‐Dmd^mdx^/J (mdx) colonies at ISU. All animals were housed and treated in standard polycarbonate cages with stainless‐steel wire bar lids and bedding (1–5 mice per cage), under thermoneutral conditions (27°C) at the ISU animal facility and maintained on a 12‐h reverse light–dark cycle. At 8 weeks of age, both C57 and mdx mice were randomly assigned to treatment: C57‐Vehicle (negative control, V), C57‐Imoxin (IMX), mdx‐V, and mdx‐IMX. Group sizes were *n* = 7–10 mice per group for muscle function tests, *n* = 6–8 mice per group for histology, and *n* = 5–6 mice per group for biochemical analyses. Fresh water and food (Envigo, Teklad global 14% protein rodent diet 2014) were provided ad libitum and replaced weekly. At 32 weeks of age, mice were anesthetized to a surgical plane with a ketamine: xylazine cocktail (80:10 mg/kg, intraperitoneal injection). While under deep anesthesia, the costal diaphragm was removed, resulting in euthanasia. One hemidiaphragm was used for in vitro muscle function analysis, and the other hemidiaphragm was divided, with one portion fixed for histological measures, and the remaining portion frozen in liquid nitrogen for biochemical measures.

### 2.2 Imoxin preparation and subcutaneous injection

Imoxin (Calbiochem; 527450, Millipore Sigma; I9785) and negative control (NC, Calbiochem; 527455) were delivered to mice through subcutaneous injection 5 days/wk. To make a stock solution, 5 mg of IMX or 10 mg of NC were reconstituted with 1 mL of 100% ethanol and stored in aliquots (20 μL IMX; 10 μL NC). For use, IMX and NC were further diluted with saline to make a working concentration of 0.1 mg/mL. Accordingly, we added 980 μL of saline into IMX tubes and 990 μL of saline into NC tubes. The volume of each injection was adjusted weekly for each mouse to deliver a dose of 0.5 mg/kg subcutaneously, as has been used previously (Li et al., 2017; Nakamura et al., 2014; Tronel et al., 2014; Valentine et al., 2018). Starting at 8 wks of age, mice were treated with vehicle or IMX, and the treatment period was approximately 24 weeks. The final dose of imoxin was given ~17–22 h before tissue collection and euthanasia.

### 2.3 Muscle function tests

At approximately 28 weeks of age, mice were transferred to the Physiological Assessment Core at the University of Florida for muscle function testing as we have done previously (Spaulding et al., 2016; Spaulding et al., 2019). Mice continued on assigned treatments, but were allowed to recover from transport for at least a 1 week prior to euthanasia. Because the mdx diaphragm mimics disease progression (Stedman et al., 1991), the costal diaphragm was collected. Following excision, in vitro muscle function tests were performed. Briefly, to measure isometric tetanic force, the diaphragm was stimulated with a frequency of 100 Hz at optimal muscle length (Lo). Specific force was determined by tetanic force normalized by estimated muscle cross sectional area (Moorwood et al., 2013). Tetanic contractions were also used to determine one‐half relaxation time (½RT) and time to peak tension. To measure dynamic and elastic passive force, muscles were stretched at 2%, 5%, 7.5%, 10%, 12.5%, and 15% of Lo with a speed of 1 Lo/s and maintained for 2 min. Peak dynamic passive force was achieved immediately after the muscle was actively stretched at a constant speed, and peak elastic passive force was the force recorded after holding the muscle at a continuous stretch for 2 min (Smith & Barton, 2014).

### 2.4 Trichrome staining

Trichrome staining was performed as previously described (Selsby et al., 2016; Spaulding et al., 2016; Spaulding et al., 2019). Briefly, 8 μm sections of OCT‐embedded diaphragms were put on slides and gently washed in PBS for 10 min. After washing, slides were placed in Bouin's solution for 18–24 h. The slides were then incubated sequentially in Weigert's hematoxylin, Biebrich scarlet acid fuchsin, PTA/PMA, and Aniline blue. Slides were dehydrated in ethanol, rinsed in Citrisolv, and sealed with nail polish. Non‐overlapping images (3–5 images/muscle) were captured at ×20 magnification using a Leica DMI3000 B microscope (Leica Microsystems, Germany) and camera (QICAM MicroPublisher 5.0 (model no. MP5.0‐ RTV‐CLR‐10; QIMAGING)) with QCapture software (Surrey, BC, Canada). Three sections per muscle were examined and one representative section with the best overall quality was selected for quantification, and 3–5 nonoverlapping images from that section were averaged to generate one value per mouse/muscle for statistical analysis. Fibrotic area was quantified by OpenLab Software (v3.5.1), and the fibrotic area was divided by the total area of muscle to determine the percent fibrosis.

### 2.5 Protein extraction and Western blot

Western blot analysis was performed as previously described (Krishna et al., 2021; Rudolph et al., 2021) with minor modifications. Briefly, the diaphragm was powdered on dry ice and protein extracted in whole muscle buffer (2% SDS, 10 mM sodium phosphate, pH 7.0) with protease inhibitor (ThermoFisher, Halt protease inhibitor cocktail, 78425), and phosphatase inhibitor (ThermoFisher, Halt phosphatase inhibitor, 78428), using a hand‐held homogenizer. Samples were centrifuged at ~10,000 × g at 4°C for 15 min, supernatant collected, and protein concentration determined using BCA (Thermo Scientific, Pierce BCA kit, 23225). Samples were prepared using a sample buffer containing Laemmli buffer (GenScript, M00676‐250,4X) and β‐mercaptoethanol (Millipore, M3148) and heated at 95°C for 5 min before storing at −80°C until subsequent use. Equal amounts of protein were loaded into each lane of 4%–20% gradient gels (Bio‐Rad, Criterion TGX Gel), protein was separated at 120–180 V for 40–60 min and transferred to nitrocellulose at 90–95 V for 1 h. Equal loading was confirmed with full lane densitometry of Ponceau‐S‐stained membranes imaged on an AzureTM c600 (AzureSpot software, Azure Biosystems, USA). Membranes were blocked in 5% nonfat dehydrated milk for 1 h in TBST (Tris‐Buffered Saline with 0.1% Tween‐20) and incubated in primary antibodies (Table 1) at 4°C overnight. After washing the membranes in TBST, membranes were incubated in secondary antibody (Fischer Scientific, 50‐195‐917, 50‐195‐914) at room temperature for 1 h. Lastly, membranes were incubated for 5 min in enhanced chemiluminescence (ECL, Bio‐Rad, 1705061) or high sensitivity ECL (ThermoFisher, 34096) under dark conditions and were imaged using AzureTM c600 and quantified using AzureSpot software.

**TABLE 1:** Western primary antibody used in the study.

Antibody	Product ID	Company	Primary (1XTBST)	Secondary (1XTBST)
ATF4	97038	Cell signaling	1:500	Ms, 1:1000 2.5% milk
ATF6	65880	Cell signaling	1:500	Rb, 1:1000 2.5% milk
BiP	3177	Cell Signaling	1:500	Rb, 1:1000 5% milk
COL1A	sc‐59772	Santa Cruz	1:1000	Ms, 1:1000 5% milk
Collagen III	ab184993	Abcam	1:1000	Rb, 1:1000 5% milk
peIF2α Ser51	3398s	Cell Signaling	1:500 2.5% milk	Rb, 1:1000 2.5% milk
eIF2α	9722	Cell Signaling	1:500	Rb, 1:1000 2.5% milk
Fibronectin	F3648	Sigma	1:2000	Rb, 1:2000
IKKα	2682	Cell signaling	1:1000	Rb, 1:1000 2.5% milk
IL1β	12,703	Cell Signaling	1:500	Rb, 1:1000 2.5% milk
pIRE1α Ser724	ab48187	Abcam	1:1000	Rb, 1:1000 2.5% milk
IRE1α	3294	Cell Signaling	1:1000	Rb, 1:1000 2.5% milk
pIκB Ser32/36	82349‐1‐RR	Proteintech	1:1000	Ms, 1:1000 5% milk
IκB	9242	Cell Signaling	1:1000	Rb, 1:2000
MyD88	4283	Cell Signaling	1:1000	Rb, 1:1000 2.5% milk
pNFκB Ser536	3033	Cell signaling	1:500	Rb, 1:2000
NFκB	8242	Cell signaling	1:1000	Rb, 1:2000
NLRP3	13,158	Cell Signaling	1:500	Rb, 1:1000 5% milk in
pPACT Ser18	sc‐53524	Santa Cruz	1:500 3% BSA	Ms, 1:1000 2.5% milk
PACT	sc‐377103	Santa Cruz	1:500 3% BSA	Ms, 1:1000 2.5% milk
pPKR Thr446	ab32036	Abcam	1:1000 3% BSA	Rb, 1:1000 2.5% milk
PKR	6282	Santa Cruz	1:1000 2.5% milk	Ms, 1:1000 2.5% milk
PP1α	sc‐271762	Santa Cruz	1:1000	Ms, 1:1000 2.5% BSA
pPERK Ser719	29546‐1‐AP	Proteintech	1:1000	Rb, 1:1000 2.5% milk
PERK	24390‐1‐AP	Proteintech	1:1000	Rb, 1:1000
TGFβ1	ab215715	Abcam	1:500	Rb, 1:500
TLR4	sc‐293072	Santa Cruz	1:1000	Ms, 1:1000 2.5% BSA
TNFα	MP390	Invitrogen	1:1000	Ms, 1:1000 2.5% milk
TRBP2	15753‐1‐AP	Proteintech	1:500	Rb, 1:500
XBP1s	40435	Cell Signaling	1:1000	Rb, 1:1000 2.5% milk

### 2.6 Statistical analysis

All data are presented as mean ± SD with significance set at *p* < 0.05. To analyze group differences in dynamic and elastic passive forces, a mixed model ANOVA was conducted in R (v4.4.1) with Tukey's post hoc tests applied for pairwise group comparisons. End‐point measures (final body weight, ½ RT, western blot, etc.) were analyzed using two‐way ANOVA with Tukey's post hoc test in GraphPad Prism 10. Data were tested for normality using Shapiro–Wilk test. When necessary to achieve a normal distribution, data were log transformed and this is indicated in figure legends. Data points more than two standard deviations from the mean were excluded from the analysis regardless of group or direction.

## 3 RESULTS

### 3.1 PKR and its regulatory proteins

**FIGURE 1:**
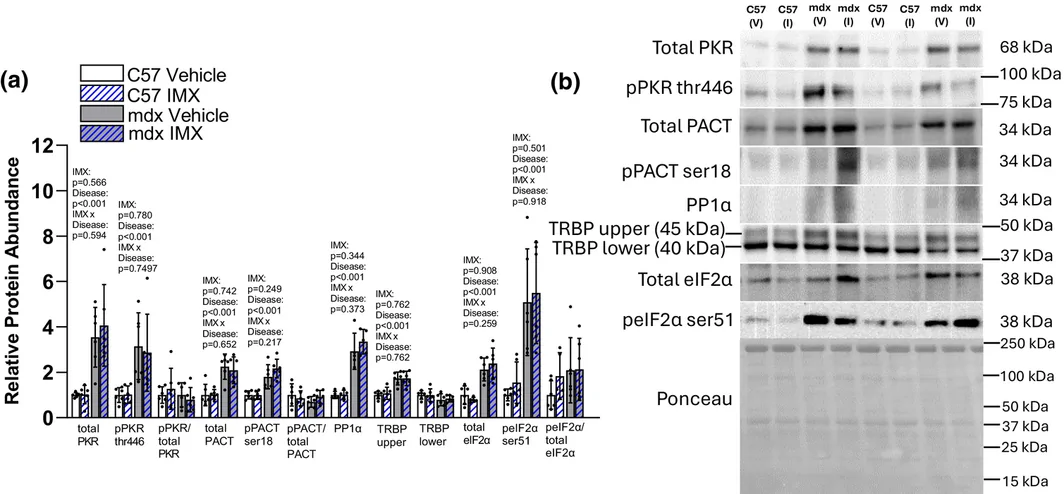
Expression of PKR and PKR‐related proteins. (a) total and pPKR, PKR regulating proteins and PKR substrates; *n* = 6/group (C57‐I for total eIF2α, eIF2α ratio, and all groups for PP1α are *n* = 5). (b) Representative western blot images and corresponding Ponceau stain image (PonS) are presented. Group differences are expressed as percent change in mdx relative to C57 vehicle (positive values indicate higher levels in mdx): PKR (254%, *p* = 0.005), pPKR (215%, *p* = 0.002), PACT (125%, *p* = 0.001), pPACT (80%, *p* = 0.005), PP1α (193%, *p* < 0.001), TRBP (72%, *p* < 0.001), eIF2α (112%, *p* = 0.004), and peIF2α (409%, *p* = 0.002). Similarly, in IMX groups, mdx‐I was elevated compared to C57‐I: PKR (300%, *p* < 0.001), pPKR (180%, *p* = 0.006), PACT (104%, *p* = 0.005), pPACT (120%, *p* < 0.001), PP1α (230%, *p* < 0.001), TRBP (61%, *p* < 0.002), eIF2α (200%, *p* < 0.001), and peIF2α (256%, *p* = 0.003). Phosphorylated PKR data were log transformed to achieve normality before statistical analysis. Data presented as mean ± SD. IMX; imoxin.

Total PKR (*p* < 0.001) and phosphorylated (p)PKR (Thr446; *p* < 0.001), as well as most PKR‐associated proteins, including total PACT (*p* < 0.001), pPACT (Ser18; *p* < 0.001), PP1α (*p* < 0.001), TRBP (45 kDa; *p* < 0.001), total eIF2α (*p* < 0.001), and peIF2α (Ser41; *p* < 0.001) were increased as a main effect of disease (all *p* < 0.05), but did not change as a main effect of IMX (Figure 1). Across both vehicle‐ and IMX‐treated conditions, mdx mice consistently showed 180%–300% higher levels of PKR and pPKR, 61%–230% higher levels of PKR regulatory proteins, and 112%–409% higher levels of eIF2α and peIF2α than C57 controls, though no significant differences were detected between mdx‐V and mdx‐I groups. Hence, this IMX dosing strategy failed to inhibit PKR phosphorylation.

### 3.2 Final body weight and muscle function

Final body weight was increased as a main effect of disease (*p* = 0.006) such that mdx groups weighed more than C57 groups but were unaffected by IMX in both C57 (*p* = 0.620) and mdx groups (*p* = 0.961). Final body weight of each group was: C57‐V (30.9 ± 2.5 g), C57‐IMX (29.1 ± 2.0 g), mdx‐V (32.5 ± 3.5 g), and mdx‐I (33.2 ± 3.3 g).

**FIGURE 2:**
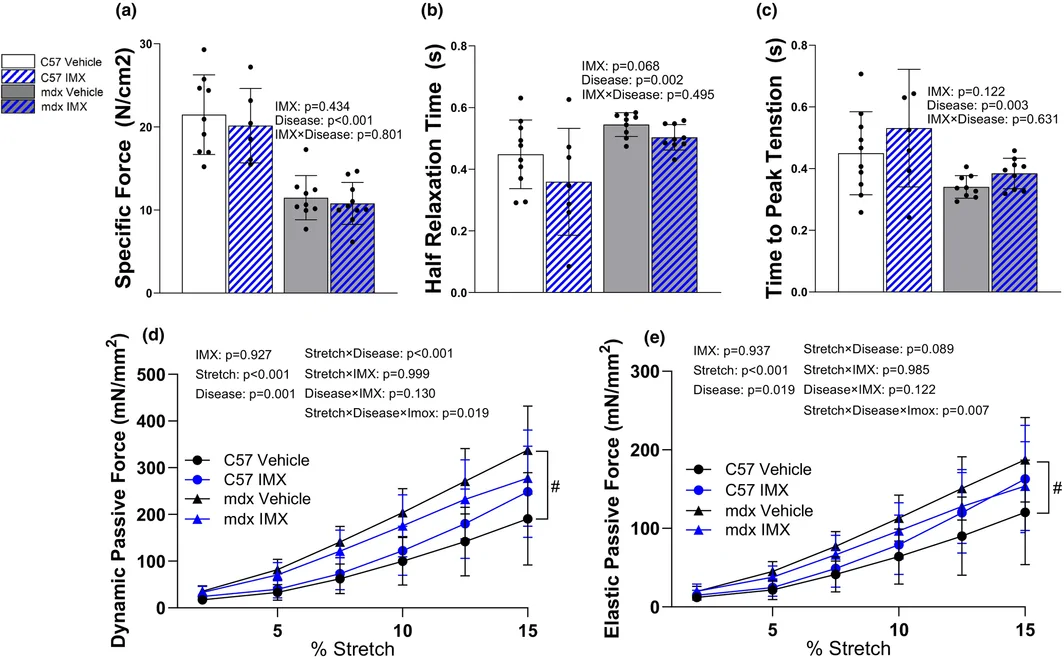
Muscle force generation. (a) Specific force was lower in mdx‐V compared to C57‐V (44%, *p* < 0.001) and in mdx‐I compared to C57‐I (46%, *p* < 0.001); C57‐V (*n* = 9), C57‐I (*n* = 6), mdx‐V (*n* = 9), mdx‐I (*n* = 10). (b) Half relaxation time (½RT, s): C57‐I relaxed 40% faster than mdx‐I (*p* = 0.039), though C57‐V was similar to mdx‐V (*p* = 0.184); C57‐V (*n* = 10), C57‐I (*n* = 7), mdx‐V (*n* = 9), and mdx‐I (*n* = 9). (c) Time to peak tension (s): C57‐V and C57‐I were similar to mdx‐V and mdx‐I, respectively; C57‐V (*n* = 10), C57‐I (*n* = 7), mdx‐V (*n* = 9), and mdx‐I (*n* = 9). (d) Dynamic passive force: Post hoc group comparisons indicated that dynamic passive force was increased in mdx‐V compared to C57‐V (*p* = 0.002), mdx‐I was similar to C57‐I (*p* = 0.417), mdx‐I was similar to mdx‐V (*p* = 0.627); C57‐V (*n* = 10), C57‐I (*n* = 7), mdx‐V (*n* = 9), and mdx‐I (*n* = 10). (e) Elastic passive force: Mdx‐V was greater than C57‐V (*p* = 0.027), mdx‐I was similar to C57‐I (*p* = 0.931), and mdx‐I was similar to mdx‐V (*p* = 0.688); C57‐V (*n* = 10), C57‐I (*n* = 7), mdx‐V (*n* = 9), and mdx‐I (*n* = 10). # Indicates pairwise post hoc group comparison between C57‐V versus mdx‐V after mixed model ANOVA (*p* < 0.05, for d, e). IMX; imoxin. Data presented as mean ± SD. For group differences in passive forces, the mixed model ANOVA was used.

Specific force was decreased (*p* < 0.001), and ½RT time (*p* = 0.002) was increased as a main effect of disease. Time to peak tension was decreased by the main effect of disease (*p* = 0.003). Imoxin did not impact specific tension, ½RT, or time to peak tension within C57 or mdx groups. Both dynamic and elastic passive forces had a main effect of stretch (both *p* < 0.001), such that passive forces rose with increased stretch (Figure 2d,e). Dynamic (*p* = 0.001) and elastic (*p* = 0.019) passive force were also increased as a main effect of disease. Furthermore, dynamic passive force had a stretch × disease (*p* < 0.001) and stretch × disease × IMX interactions (*p* = 0.019), whereas elastic passive force showed a stretch × disease × IMX interaction (*p* = 0.007).

### 3.3 Percent fibrosis and fibrosis‐associated proteins

**FIGURE 3:**
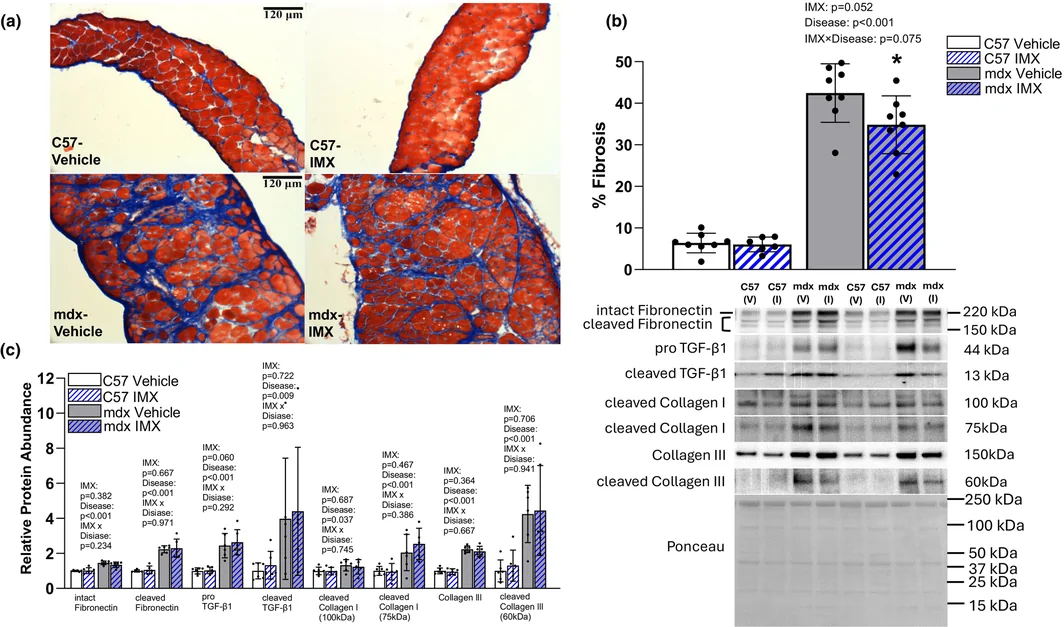
Dystrophic diaphragm fibrosis and fibrosis regulating proteins. (a) Representative stained images of C57‐Vehicle, C57‐IMX, mdx‐Vehicle, and mdx‐IMX. (b) Percent fibrosis; *n* = 6/group (C57‐V for cleaved fibronectin, mdx‐I for cleaved col. I (75 kDa, 100 kDa) are *n* = 5), (c) fibrosis regulating proteins; *n* = 5‐6/group. Representative western blot images and corresponding Ponceau stain image (PonS) are presented. Group differences are expressed as percent change in mdx relative to C57 (positive values indicate higher levels in mdx): Percent fibrosis (564%, *p* < 0.001), intact fibronectin (47%, *p* < 0.001), cleaved fibronectin (134%, *p* < 0.001), pro TGF‐β1 (503%, *p* < 0.001), cleaved TGF‐β1 (298%, *p* = 0.036), cleaved Collagen I (100 kDa, *p* = 0.276), cleaved Collagen I (75 kDa, *p* = 0.090), Collagen III (123%, *p* < 0.001), and cleaved Collagen III (325%, *p* = 0.012). Similarly, in IMX groups, mdx‐I was elevated compared to C57‐I: Percent fibrosis (477%, *p* < 0.001), intact fibronectin (33%, *p* = 0.001), cleaved fibronectin (118%, *p* < 0.001), pro TGF‐β1 (156%, *p* < 0.001), cleaved TGF‐β1 (230%, *p* = 0.071), cleaved Collagen I (75 kDa, 166%, *p* = 0.009), cleaved Collagen I (100 kDa, *p* = 0.561), Collagen III (120%, *p* < 0.001), and cleaved Collagen III (242%, *p* = 0.015). * Indicates difference compared to mdx‐V (*p* < 0.05). Cleaved TGF‐β1 data were log transformed before statistical analysis. Data presented as mean ± SD. IMX; imoxin.

Percent fibrosis (*p* < 0.001) was increased as a main effect of disease (Figure 3) and was increased in mdx‐V compared to C57‐V (564%, *p* < 0.001) and in mdx‐I compared to C57‐I (477%, *p* < 0.001). Of note, percent fibrosis was reduced 18% by IMX in mdx mice (*p* = 0.038), raising the possibility of a therapeutic role of IMX. Relative protein abundance of fibrosis‐associated proteins, fibronectin (*p* < 0.001), pro TGF‐β1 (*p* < 0.001), cleaved TGF‐β1 (*p* < 0.001), along with the most abundant collagen types (I and III) in muscle (McKee et al., 2019), cleaved Collagen I (100 kDa, *p* = 0.037), cleaved Collagen I (75 kDa, *p* < 0.001), Collagen III (*p* < 0.001), and cleaved Collagen III (*p* = 0.005) were increased as a main effect of disease such that mdx groups were greater than C57 groups (Figure 2). Across both vehicle‐ and IMX‐treated conditions, mdx mice consistently showed 477%–564% greater fibrosis, 33%–134% higher levels of intact and cleaved fibronectin, 156%–503% higher levels of pro‐TGF‐β1, and 120%–325% higher levels of collagen III and cleaved collagen III than respective C57 groups. Cleaved TGF‐β1 was 230%–298% higher in mdx mice, although the difference was significant only under vehicle‐treated conditions. Collagen I cleavage products were generally not significant among groups, except for the 75‐kDa fragment, which was 166% higher in mdx‐I than C57‐I. Finally, relative protein abundance of fibronectin, TGF‐β1, and collagen proteins were similar in mdx‐I compared to mdx‐V. Overall, despite lowering percent fibrotic area, IMX did not impact relative protein abundance of fibrosis‐associated proteins.

### 3.4 ER stress associated proteins

**FIGURE 4:**
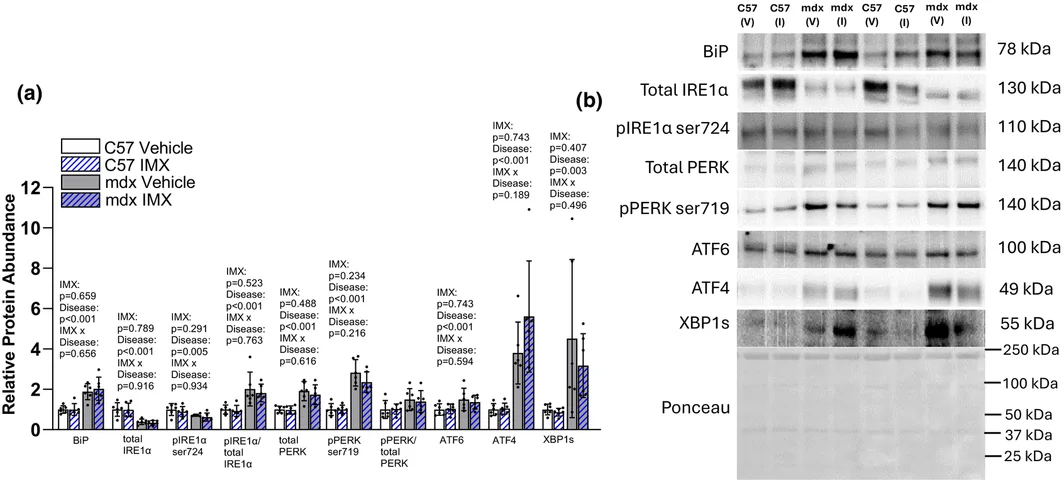
Expression of ER stress‐associated proteins. (a) ER stress associated proteins; *n* = 6/group. (b) Representative western blot images and corresponding Ponceau stain image (PonS) are presented. Group differences are expressed as percent change in mdx relative to C57 (positive values indicate higher levels in mdx). In vehicle groups, BiP (88%, *p* < 0.01), total PERK (91%, *p* < 0.01), pPERK (182%, *p* < 0.001), total IRE1α (−61%, *p* = 0.002), pIRE1α (*p* = 0.138), pIRE1α /IRE1α (102%, *p* = 0.011), ATF6 (*p* = 0.130), ATF4 (280%, *p* < 0.001), and XBP1s (350%, *p* = 0.009). Similarly, in IMX groups, mdx‐I was elevated compared to C57‐I: BiP (103%, *p* = 0.001), total PERK (79%, *p* < 0.01), pPERK (134%, *p* < 0.01), total IRE1α (−65%, *p* = 0.002), pIRE1α (*p* = 0.170), pIRE1α /IRE1α (96%, *p* = 0.030), ATF6 (*p* = 0.434), ATF4 (438%, *p* < 0.001), and XBP1s (266%, *p* < 0.008). ATF4, XBP1s data were log transformed before statistical analysis. Data presented as mean ± SD. IMX; imoxin.

When we considered ER stress proteins, we discovered that BiP (*p* < 0.001), pIRE1α/IRE1α (*p* < 0.001), total PERK (*p* < 0.001), pPERK (Ser719; *p* < 0.001), ATF6 (*p* = 0.013), ATF4 (*p* < 0.001), and XBP1s (*p* = 0.003) were increased as a main effect of disease, whereas pIRE1α (Ser724; *p* = 0.005) and total IRE1α (*p* < 0.001) were decreased as a main effect of disease (Figure 4). Across both vehicle‐ and IMX‐treated conditions, mdx mice consistently had 88%–103% higher levels of BiP, 79%–182% higher levels of total and phosphorylated PERK, 280%–438% higher ATF4, and 266%–350% higher XBP1s than respective C57. Total IRE1α was 61%–65% lower in mdx mice, whereas the pIRE1α/IRE1α ratio was 96%–102% higher than respective C57 mice. In contrast, absolute pIRE1α and ATF6 levels did not differ significantly between mdx and C57 mice under either treatment condition. Notably, relative protein abundance of all ER stress‐related proteins was similar between mdx‐V and mdx‐I.

### 3.5 Inflammatory signaling proteins

**FIGURE 5:**
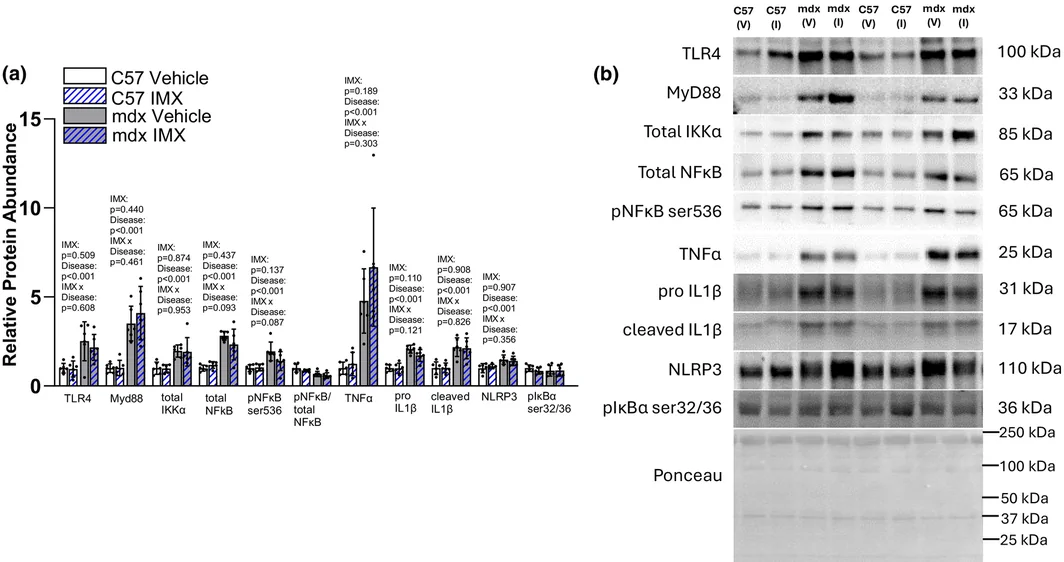
Expression of inflammatory signaling proteins. (a) Inflammatory signaling proteins; *n* = 6/group (C57‐I for NLRP3 is *n* = 5). (b) Representative western blot images and corresponding Ponceau stain image (PonS) are presented. Group differences are expressed as percent change in mdx relative to C57 (positive values indicate higher levels in mdx). In vehicle groups, TLR4 (151%, *p* = 0.007)), MyD88 (250%, *p* < 0.001), total IKKα (95%, *p* = 0.012), total NFκB (182%, *p* < 0.001), pNFκB (95%, *p* = 0.003), TNFα (378%, *p* = 0.014), pro‐IL1β (107%, *p* < 0.001), cleaved‐IL1β (118%, *p* = 0.002), NLRP3 (48%, *p* = 0.011), and pIκBα (*p* = 0.751). Similarly, in IMX groups, mdx‐I was elevated compared to C57‐I: TLR4 (126%, *p* = 0.034), MyD88 (304%, *p* < 0.001), total IKKα (96%, *p* < 0.006), total NFκB (97%, *p* = 0.002), pNFκB (35%, *p* = 0.421), TNFα (439%, *p* < 0.001), pro‐IL1β (72%, *p* < 0.001), cleaved‐IL1β (108%, *p* = 0.002). NLRP3 (*p* = 0.180), and pIκBα (*p* = 0.999). Total IKKα, pIκBα, and TNFα data were log transformed before statistical analysis. Data presented as mean ± SD. IMX; imoxin.

Increased inflammatory signaling is a hallmark of dystrophic muscle injury (Chen et al., 2005; Duan et al., 2021; Monici et al., 2003). Here, there was a main effect of disease on inflammatory signaling proteins, TLR4 (*p* < 0.001), MyD88 (*p* < 0.001), total IKKα (*p* < 0.001), total NFκB (*p* < 0.001), pNFκB (Ser536; *p* < 0.001), TNFα (*p* < 0.001), pro IL1β (*p* < 0.001), cleaved IL1β (*p* < 0.001), and NLRP3 (*p* < 0.001) as mdx groups were higher than C57 groups (Figure 5). Across both vehicle‐ and IMX‐treated conditions, mdx mice consistently showed 126%–151% higher levels of TLR4, 250%–304% higher levels of MyD88, 95%–96% higher levels of total IKKα, 97%–182% higher levels of total NFκB, 378%–439% higher levels of TNFα, and 72%–118% higher levels of pro‐ and cleaved IL‐1β than corresponding C57 groups. Phosphorylated NFκB was 35%–95% higher in mdx mice, although the difference was significant only under vehicle‐treated conditions. NLRP3 was 48% higher in mdx‐V than C57‐V but did not differ significantly between the IMX‐treated groups, whereas pIκBα did not differ significantly between mdx and C57 mice under either treatment condition. All assessed inflammatory signaling proteins were similar between mdx‐V and mdx‐I.

## 4 DISCUSSION

DMD is a fatal, X‐linked disease characterized by the absence of dystrophin, leading to progressive muscle degeneration, impaired muscle function, and ultimately death (Duan et al., 2021; Mah et al., 2014). We recently reported that a major integrated stress response kinase, PKR, is elevated in dystrophic skeletal muscle (Krishna, Echevarria, et al., 2023; Krishna, Spaulding, et al., 2023). Importantly, PKR is implicated in ER stress (Eo & Valentine, 2021; Eo & Valentine, 2022; Gal‐Ben‐Ari et al., 2018; Shimazawa et al., 2007), apoptosis (Lee et al., 2007; Singh et al., 2009), and inflammatory signaling (Bonnet et al., 2000; Chukwurah et al., 2021; Goh et al., 2000; Lu et al., 2012), all of which are dysregulated in dystrophic muscles (Duan et al., 2021; Haslett et al., 2002; Krishna, Spaulding, et al., 2023; Moorwood & Barton, 2014). These findings highlight PKR as a potential therapeutic node that can be targeted to slow disease progression. In this study, we discovered that long‐term treatment with IMX failed to reduce PKR phosphorylation or preserve muscle function and did not decrease inflammatory signaling or ER stress‐associated proteins in the dystrophic diaphragm. Despite the failure of IMX to inhibit PKR phosphorylation, consistent upregulation of PKR alongside inflammatory and ER stress markers, as well as their interconnected relationship, continue to emphasize PKR as a potential central regulatory node in DMD.

Imoxin blocks PKR autophosphorylation, thereby inhibiting its catalytic activity (Jammi et al., 2003). More subtly, IMX appears to block autophosphorylation of inactivated PKR, but may not remove existing phosphorylation of PKR (Jammi et al., 2003). The treatment paradigm used in this study was similar to those used in previous investigations. One month of IMX treatment, using the same dose and delivery approach as used herein (0.5 mg/kg; subcutaneous, daily), effectively decreased PKR activation in a mouse model of obesity (Nakamura et al., 2014). Acutely, IMX treatment decreased LPS‐mediated elevations in PKR phosphorylation as well as inflammatory and atrophy transcripts in mouse skeletal muscle (Valentine et al., 2018), and also decreased PKR phosphorylation in rat models of neuroinflammation (Tronel et al., 2014; Xiao et al., 2016). Further, more prolonged IMX treatment studies have shown that 4 wks of daily intraperitoneal (IP) injection of IMX (0.5 mg/kg) effectively attenuated PKR protein expression and inflammatory markers in a rat hypertension model (Kalra et al., 2020), and 5 days of daily high dose IMX (5 mg/kg; subcutaneous) attenuated PKR phosphorylation and muscle loss in a mouse model of cancer cachexia (Eley et al., 2007). Long‐term intraperitoneal IMX delivery also effectively reduced PKR phosphorylation and inflammation in a mouse lung injury model (Li et al., 2017). In vitro studies further support the efficacy of IMX in inhibiting PKR activity. Importantly, we and others demonstrated that IMX reduced palmitate‐ (Mangali et al., 2019), tunicamycin‐ (Eo & Valentine, 2021), and dexamethasone‐ (Eo et al., 2020) induced PKR phosphorylation in C2C12 cells and palmitate‐induced inflammatory signaling in cardiomyocytes (Eo & Valentine, 2022). Given this record of IMX‐mediated reduction in PKR activity and subsequent inhibition of downstream signaling, the persistence of PKR activity in the present investigation is surprising. Despite previous PKR inhibition using a similar dose or delivery strategy (Kalra et al., 2020; Nakamura et al., 2014), inhibition of PKR in the context of dystrophin deficiency may require a higher dose of IMX than used here. Indeed, in a cachexia model, a high dose of IMX (5 mg/kg) prevented muscle loss, whereas a lower dose (1 mg/kg) did not (Eley et al., 2007), raising the possibility of a therapeutic threshold in certain biological contexts.

As chronic muscle injury and cellular dysfunctions progressively worsen in the dystrophic diaphragm, the intracellular environment may promote sustained PKR activation. For example, increased inflammation is already present in the diaphragm of 4‐week‐old mdx mice (Howard et al., 2021) and persists with advancing age (Mojumdar et al., 2014), and is a potent activator of PACT, an upstream PKR kinase. Further, PACT can be activated in response to ER stress (Singh et al., 2009) or oxidative stress (Patel et al., 2000), as is common in DMD (Hulmi et al., 2016; Krishna, Spaulding, et al., 2023; Moorwood & Barton, 2014; Pauly et al., 2017; Selsby, 2011), even in the absence of double‐stranded RNA (Li et al., 2006). In the data presented herein, dystrophin deficiency resulted in a robust increase in p‐ and total PACT that is likely activating PKR. Surprisingly, two potent inhibitors of PKR phosphorylation, TRBP and PP1α, were also increased by dystrophin deficiency. Hence, it is apparent that dystrophic muscle is differentially regulating PKR; however, the net sum of regulation is likely to support PKR activation, akin to the difficulty of pharmacological inhibition of calpain due to its constitutively increased activity in dystrophic muscles (Selsby et al., 2010; Spencer et al., 1995).

Consistent with previous reports, specific tension and time to peak tension were decreased (Hakim et al., 2019; Selsby et al., 2010; Spaulding et al., 2016) and 1/2RT was increased by disease (Dupont‐Versteegden & McCarter, 1992; Hakim et al., 2019). These contractile changes are due, at least in part, to the selective loss of type 2 fibers causing a type I shift (Pedemonte et al., 1999; Petrof et al., 1993) and loss of calcium homeostatic control (Mareedu et al., 2021). We also demonstrated that dynamic and elastic passive forces increased by disease in this study, consistent with previous findings (Brashear et al., 2022; Loehr et al., 2018). These changes in passive force are supportive of increased stiffness and are likely driven, at least in part, through altered collagen structure (Brashear et al., 2021; Smith & Barton, 2014). While the percent fibrosis was increased by disease and was partially corrected by IMX, IMX did not coordinately reduce relative protein abundance of fibrosis‐related proteins. The discrepancy of histological data and immunoblot data could be due to methodological differences, as trichrome staining measures collagen fibers in the extracellular matrix rather than detecting soluble collagens like with Western blot (Zadvornyi et al., 2023). Finally, we cannot exclude the beneficial effects of IMX on this metric of fibrosis through a PKR‐independent mechanism (Chen et al., 2008), particularly given the totality of the data contained herein.

In total, this IMX dosing regimen failed to effectively decrease PKR phosphorylation or attenuate disease severity in mdx mice. Nevertheless, given its role as a key signaling protein, PKR remains an interesting therapeutic candidate. The intracellular environment seems poised to maintain PKR activation given persistent inflammation and ER stress. Indeed, the complex regulation of PKR activation and inhibition could make it difficult to inhibit PKR in dystrophic muscle. Preventing the ability of activators to interact with PKR or enhancing its endogenous inhibitors may convey a better therapeutic effect than targeting autophosphorylation using IMX.

## AUTHOR CONTRIBUTIONS

**Ji Heun Lee:** Conceptualization; data curation; formal analysis; investigation; methodology; visualization. **Morgan E. Vorwald:** Investigation; methodology. **Melissa Roths:** Investigation; methodology. **Joshua T. Selsby:** Conceptualization; formal analysis; funding acquisition; investigation; methodology; project administration; resources; supervision; visualization. **Rudy J. Valentine:** Conceptualization; formal analysis; funding acquisition; investigation; methodology; project administration; resources; supervision; visualization.

## FUNDING INFORMATION

This work was supported by NIH grant NIH R21 AR080362 (RJV and JTS).

## ETHICS STATEMENT

All animal work was approved by the IACUC at Iowa State University (ISU; IACUC‐23‐110) and the University of Florida (202108822).

## Data Availability

The data that support the findings of this study are available from the corresponding author upon reasonable request.

## References

[phy271115-bib-0001] Birnkrant, D. J. , Bushby, K. , Bann, C. M. , Apkon, S. D. , Blackwell, A. , Brumbaugh, D. , Case, L. E. , Clemens, P. R. , Hadjiyannakis, S. , Pandya, S. , Street, N. , Tomezsko, J. , Wagner, K. R. , Ward, L. M. , Weber, D. R. , & DMD Care Considerations Working Group . (2018). Diagnosis and management of Duchenne muscular dystrophy, part 1: Diagnosis, and neuromuscular, rehabilitation, endocrine, and gastrointestinal and nutritional management. Lancet Neurology, 17(3), 251–267. 10.1016/S1474-4422(18)30024-3 PMC5869704 29395989

[phy271115-bib-0002] Bonnet, M. C. , Weil, R. , Dam, E. , Hovanessian, A. G. , & Meurs, E. F. (2000). PKR stimulates NF‐kappaB irrespective of its kinase function by interacting with the IkappaB kinase complex. Molecular and Cellular Biology, 20(13), 4532–4542. 10.1128/mcb.20.13.4532-4542.2000 PMC85837 10848580

[phy271115-bib-0003] Brashear, S. E. , Wohlgemuth, R. P. , Gonzalez, G. , & Smith, L. R. (2021). Passive stiffness of fibrotic skeletal muscle in mdx mice relates to collagen architecture. The Journal of Physiology, 599(3), 943–962. 10.1113/JP280656 PMC9926974 33247944

[phy271115-bib-0004] Brashear, S. E. , Wohlgemuth, R. P. , Hu, L. Y. , Jbeily, E. H. , Christiansen, B. A. , & Smith, L. R. (2022). Collagen cross‐links scale with passive stiffness in dystrophic mouse muscles, but are not altered with administration of a lysyl oxidase inhibitor. PLoS One, 17(10), e0271776. 10.1371/journal.pone.0271776 PMC9612445 36302059

[phy271115-bib-0005] Carvalho, B. M. , Oliveira, A. G. , Ueno, M. , Araújo, T. G. , Guadagnini, D. , Carvalho‐Filho, M. A. , Geloneze, B. , Lima, M. M. O. , Pareja, J. C. , Carvalheira, J. B. C. , & Saad, M. J. A. (2013). Modulation of double‐stranded RNA‐activated protein kinase in insulin sensitive tissues of obese humans. Obesity (Silver Spring), 21(12), 2452–2457. 10.1002/oby.20410 23519983

[phy271115-bib-0006] Chen, H. M. , Wang, L. , & D'Mello, S. R. (2008). A chemical compound commonly used to inhibit PKR, 8‐(imidazol‐4‐ylmethylene)‐6H‐azolidino[5,4‐g] benzothiazol‐7‐one, protects neurons by inhibiting cyclin‐dependent kinase. European Journal of Neuroscience, 28(10), 2003–2016. 10.1111/j.1460-9568.2008.06491.x PMC3320856 19046382

[phy271115-bib-0007] Chen, Y. W. , Nagaraju, K. , Bakay, M. , McIntyre, O. , Rawat, R. , Shi, R. , & Hoffman, E. P. (2005). Early onset of inflammation and later involvement of TGFbeta in Duchenne muscular dystrophy. Neurology, 65(6), 826–834. 10.1212/01.wnl.0000173836.09176.c4 16093456

[phy271115-bib-0008] Chukwurah, E. , Farabaugh, K. T. , Guan, B. J. , Ramakrishnan, P. , & Hatzoglou, M. (2021). A tale of two proteins: PACT and PKR and their roles in inflammation. The FEBS Journal, 288(22), 6365–6391. 10.1111/febs.15691 PMC9248962 33387379

[phy271115-bib-0009] Costa‐Mattioli, M. , & Walter, P. (2020). The integrated stress response: From mechanism to disease. Science, 368(6489), eaat5314. 10.1126/science.aat5314 PMC8997189 32327570

[phy271115-bib-0010] Duan, D. , Goemans, N. , Takeda, S.’. , Mercuri, E. , & Aartsma‐Rus, A. (2021). Duchenne muscular dystrophy. Nature Reviews. Disease Primers, 7(1), 13. 10.1038/s41572-021-00248-3 PMC10557455 33602943

[phy271115-bib-0011] Dupont‐Versteegden, E. E. , & McCarter, R. J. (1992). Differential expression of muscular dystrophy in diaphragm versus hindlimb muscles of mdx mice. Muscle & Nerve, 15(10), 1105–1110. 10.1002/mus.880151008 1406767

[phy271115-bib-0012] Eley, H. L. , Russell, S. T. , & Tisdale, M. J. (2007). Attenuation of muscle atrophy in a murine model of cachexia by inhibition of the dsRNA‐dependent protein kinase. British Journal of Cancer, 96(8), 1216–1222. 10.1038/sj.bjc.6603704 PMC2360141 17387345

[phy271115-bib-0013] Eo, H. , Reed, C. H. , & Valentine, R. J. (2020). Imoxin prevents dexamethasone‐induced promotion of muscle‐specific E3 ubiquitin ligases and stimulates anabolic signaling in C2C12 myotubes. Biomedicine & Pharmacotherapy, 128, 110238. 10.1016/j.biopha.2020.110238 32450522

[phy271115-bib-0014] Eo, H. , & Valentine, R. J. (2021). Imoxin inhibits tunicamycin‐induced endoplasmic reticulum stress and restores insulin signaling in C2C12 myotubes. American Journal of Physiology. Cell Physiology, 321(2), C221–C229. 10.1152/ajpcell.00544.2020 34077277

[phy271115-bib-0015] Eo, H. , & Valentine, R. J. (2022). Saturated fatty acid‐induced endoplasmic reticulum stress and insulin resistance are prevented by Imoxin in C2C12 myotubes. Frontiers in Physiology, 13, 842819. 10.3389/fphys.2022.842819 PMC9355746 35936891

[phy271115-bib-0016] Gal‐Ben‐Ari, S. , Barrera, I. , Ehrlich, M. , & Rosenblum, K. (2018). PKR: A kinase to remember. Frontiers in Molecular Neuroscience, 11, 480. 10.3389/fnmol.2018.00480 PMC6333748 30686999

[phy271115-bib-0017] Goh, K. C. , deVeer, M. J. , & Williams, B. R. (2000). The protein kinase PKR is required for p38 MAPK activation and the innate immune response to bacterial endotoxin. EMBO Journal, 19(16), 4292–4297. 10.1093/emboj/19.16.4292 PMC302024 10944112

[phy271115-bib-0018] Hakim, C. H. , Lessa, T. B. , Jenkins, G. J. , Yang, N. N. , Ambrosio, C. E. , & Duan, D. (2019). An improved method for studying mouse diaphragm function. Scientific Reports, 9(1), 19453. 10.1038/s41598-019-55704-8 PMC6923406 31857625

[phy271115-bib-0019] Haslett, J. N. , Sanoudou, D. , Kho, A. T. , Bennett, R. R. , Greenberg, S. A. , Kohane, I. S. , Beggs, A. H. , & Kunkel, L. M. (2002). Gene expression comparison of biopsies from Duchenne muscular dystrophy (DMD) and normal skeletal muscle. Proceedings of the National Academy of Sciences of the United States of America, 99(23), 15000–15005. 10.1073/pnas.192571199 PMC137534 12415109

[phy271115-bib-0020] Howard, Z. M. , Lowe, J. , Blatnik, A. J., 3rd , Roberts, D. , Burghes, A. H. M. , Bansal, S. S. , & Rafael‐Fortney, J. A. (2021). Early inflammation in muscular dystrophy differs between limb and respiratory muscles and increases with dystrophic severity. The American Journal of Pathology, 191(4), 730–747. 10.1016/j.ajpath.2021.01.008 PMC8147105 33497702

[phy271115-bib-0021] Hugon, J. , Mouton‐Liger, F. , Dumurgier, J. , & Paquet, C. (2017). PKR involvement in Alzheimer's disease. Alzheimer's Research & Therapy, 9(1), 83. 10.1186/s13195-017-0308-0 PMC5629792 28982375

[phy271115-bib-0022] Hulmi, J. J. , Hentilä, J. , DeRuisseau, K. C. , Oliveira, B. M. , Papaioannou, K. G. , Autio, R. , Kujala, U. M. , Ritvos, O. , Kainulainen, H. , Korkmaz, A. , & Atalay, M. (2016). Effects of muscular dystrophy, exercise and blocking activin receptor IIB ligands on the unfolded protein response and oxidative stress. Free Radical Biology & Medicine, 99, 308–322. 10.1016/j.freeradbiomed.2016.08.017 27554968

[phy271115-bib-0023] Jammi, N. V. , Whitby, L. R. , & Beal, P. A. (2003). Small molecule inhibitors of the RNA‐dependent protein kinase. Biochemical and Biophysical Research Communications, 308(1), 50–57. 10.1016/s0006-291x(03)01318-4 12890478

[phy271115-bib-0024] Kalra, J. , Dasari, D. , Bhat, A. , Mangali, S. , Goyal, S. G. , Jadhav, K. B. , & Dhar, A. (2020). PKR inhibitor imoxin prevents hypertension, endothelial dysfunction and cardiac and vascular remodelling in L‐NAME‐treated rats. Life Sciences, 262, 118436. 10.1016/j.lfs.2020.118436 32950570

[phy271115-bib-0025] Krishna, S. , Echevarria, K. G. , Reed, C. H. , Eo, H. , Wintzinger, M. , Quattrocelli, M. , Valentine, R. J. , & Selsby, J. T. (2023). A fat‐ and sucrose‐enriched diet causes metabolic alterations in mdx mice. American Journal of Physiology. Regulatory, Integrative and Comparative Physiology, 325(6), R692–R711. 10.1152/ajpregu.00246.2022 PMC11178302 37811713

[phy271115-bib-0026] Krishna, S. , Spaulding, H. R. , Koltes, J. E. , Quindry, J. C. , Valentine, R. J. , & Selsby, J. T. (2023). Indicators of increased ER stress and UPR in aged D2‐mdx and human dystrophic skeletal muscles. Frontiers in Physiology, 14, 1152576. 10.3389/fphys.2023.1152576 PMC10166835 37179835

[phy271115-bib-0027] Krishna, S. , Spaulding, H. R. , Quindry, T. S. , Hudson, M. B. , Quindry, J. C. , & Selsby, J. T. (2021). Indices of defective autophagy in whole muscle and lysosome enriched fractions from aged D2‐mdx mice. Frontiers in Physiology, 12, 691245. 10.3389/fphys.2021.691245 PMC8299564 34305644

[phy271115-bib-0028] Landfeldt, E. , Thompson, R. , Sejersen, T. , McMillan, H. J. , Kirschner, J. , & Lochmüller, H. (2020). Life expectancy at birth in Duchenne muscular dystrophy: A systematic review and meta‐analysis. European Journal of Epidemiology, 35(7), 643–653. 10.1007/s10654-020-00613-8 PMC7387367 32107739

[phy271115-bib-0029] Lee, E. S. , Yoon, C. H. , Kim, Y. S. , & Bae, Y. S. (2007). The double‐strand RNA‐dependent protein kinase PKR plays a significant role in a sustained ER stress‐induced apoptosis. FEBS Letters, 581(22), 4325–4332. 10.1016/j.febslet.2007.08.001 17716668

[phy271115-bib-0030] Li, S. , Peters, G. A. , Ding, K. , Zhang, X. , Qin, J. , & Sen, G. C. (2006). Molecular basis for PKR activation by PACT or dsRNA. Proceedings of the National Academy of Sciences of the United States of America, 103(26), 10005–10010. 10.1073/pnas.0602317103 PMC1502496 16785445

[phy271115-bib-0031] Li, Y. , Xiao, J. , Tan, Y. , Wang, J. , Zhang, Y. , Deng, X. , & Luo, Y. (2017). Inhibition of PKR ameliorates lipopolysaccharide‐induced acute lung injury by suppressing NF‐kappaB pathway in mice. Immunopharmacology and Immunotoxicology, 39(4), 165–172. 10.1080/08923973.2017.1303839 28511573

[phy271115-bib-0032] Loehr, J. A. , Wang, S. , Cully, T. R. , Pal, R. , Larina, I. V. , Larin, K. V. , & Rodney, G. G. (2018). NADPH oxidase mediates microtubule alterations and diaphragm dysfunction in dystrophic mice. eLife, 7, 7. 10.7554/eLife.31732 PMC5812717 29381135

[phy271115-bib-0033] Lu, B. , Nakamura, T. , Inouye, K. , Li, J. , Tang, Y. , Lundbäck, P. , Valdes‐Ferrer, S. I. , Olofsson, P. S. , Kalb, T. , Roth, J. , Zou, Y. , Erlandsson‐Harris, H. , Yang, H. , Ting, J. P. Y. , Wang, H. , Andersson, U. , Antoine, D. J. , Chavan, S. S. , Hotamisligil, G. S. , & Tracey, K. J. (2012). Novel role of PKR in inflammasome activation and HMGB1 release. Nature, 488(7413), 670–674. 10.1038/nature11290 PMC4163918 22801494

[phy271115-bib-0034] Mah, J. K. , Korngut, L. , Dykeman, J. , Day, L. , Pringsheim, T. , & Jette, N. (2014). A systematic review and meta‐analysis on the epidemiology of Duchenne and Becker muscular dystrophy. Neuromuscular Disorders, 24(6), 482–491. 10.1016/j.nmd.2014.03.008 24780148

[phy271115-bib-0035] Mangali, S. , Bhat, A. , Udumula, M. P. , Dhar, I. , Sriram, D. , & Dhar, A. (2019). Inhibition of protein kinase R protects against palmitic acid‐induced inflammation, oxidative stress, and apoptosis through the JNK/NF‐kB/NLRP3 pathway in cultured H9C2 cardiomyocytes. Journal of Cellular Biochemistry, 120(3), 3651–3663. 10.1002/jcb.27643 30259999

[phy271115-bib-0036] Marchal, J. A. , Lopez, G. J. , Peran, M. , Comino, A. , Delgado, J. R. , García‐García, J. A. , Conde, V. , Aranda, F. M. , Rivas, C. , Esteban, M. , & Garcia, M. A. (2014). The impact of PKR activation: From neurodegeneration to cancer. The FASEB Journal, 28(5), 1965–1974. 10.1096/fj.13-248294 24522206

[phy271115-bib-0037] Mareedu, S. , Million, E. D. , Duan, D. , & Babu, G. J. (2021). Abnormal calcium handling in Duchenne muscular dystrophy: Mechanisms and potential therapies. Frontiers in Physiology, 12, 647010. 10.3389/fphys.2021.647010 PMC8063049 33897454

[phy271115-bib-0038] Martinez, N. W. , Gomez, F. E. , & Matus, S. (2021). The potential role of protein kinase R as a regulator of age‐related neurodegeneration. Frontiers in Aging Neuroscience, 13, 638208. 10.3389/fnagi.2021.638208 PMC8113420 33994991

[phy271115-bib-0039] McKee, T. J. , Perlman, G. , Morris, M. , & Komarova, S. V. (2019). Extracellular matrix composition of connective tissues: A systematic review and meta‐analysis. Scientific Reports, 9(1), 10542. 10.1038/s41598-019-46896-0 PMC6646303 31332239

[phy271115-bib-0040] McKim, D. A. , Griller, N. , LeBlanc, C. , Woolnough, A. , & King, J. (2013). Twenty‐four hour noninvasive ventilation in Duchenne muscular dystrophy: A safe alternative to tracheostomy. Canadian Respiratory Journal, 20(1), e5–e9. 10.1155/2013/406163 PMC3628652 23457679

[phy271115-bib-0041] Meurs, E. , Chong, K. , Galabru, J. , Thomas, N. S. B. , Kerr, I. M. , Williams, B. R. G. , & Hovanessian, A. G. (1990). Molecular cloning and characterization of the human double‐stranded RNA‐activated protein kinase induced by interferon. Cell, 62(2), 379–390. 10.1016/0092-8674(90)90374-n 1695551

[phy271115-bib-0042] Mojumdar, K. , Liang, F. , Giordano, C. , Lemaire, C. , Danialou, G. , Okazaki, T. , Bourdon, J. , Rafei, M. , Galipeau, J. , Divangahi, M. , & Petrof, B. J. (2014). Inflammatory monocytes promote progression of Duchenne muscular dystrophy and can be therapeutically targeted via CCR2. EMBO Molecular Medicine, 6(11), 1476–1492. 10.15252/emmm.201403967 PMC4237472 25312642

[phy271115-bib-0043] Monici, M. C. , Aguennouz, M. , Mazzeo, A. , Messina, C. , & Vita, G. (2003). Activation of nuclear factor‐kappaB in inflammatory myopathies and Duchenne muscular dystrophy. Neurology, 60(6), 993–997. 10.1212/01.wnl.0000049913.27181.51 12654966

[phy271115-bib-0044] Moorwood, C. , & Barton, E. R. (2014). Caspase‐12 ablation preserves muscle function in the mdx mouse. Human Molecular Genetics, 23(20), 5325–5341. 10.1093/hmg/ddu249 PMC4168821 24879640

[phy271115-bib-0045] Moorwood, C. , Liu, M. , Tian, Z. , & Barton, E. R. (2013). Isometric and eccentric force generation assessment of skeletal muscles isolated from murine models of muscular dystrophies. Journal of Visualized Experiments, 71, e50036. 10.3791/50036 PMC3596910 23407283

[phy271115-bib-0046] Nakamura, T. , Arduini, A. , Baccaro, B. , Furuhashi, M. , & Hotamisligil, G. S. (2014). Small‐molecule inhibitors of PKR improve glucose homeostasis in obese diabetic mice. Diabetes, 63(2), 526–534. 10.2337/db13-1019 PMC3900541 24150608

[phy271115-bib-0047] Nakamura, T. , Furuhashi, M. , Li, P. , Cao, H. , Tuncman, G. , Sonenberg, N. , Gorgun, C. Z. , & Hotamisligil, G. S. (2010). Double‐stranded RNA‐dependent protein kinase links pathogen sensing with stress and metabolic homeostasis. Cell, 140(3), 338–348. 10.1016/j.cell.2010.01.001 PMC2820414 20144759

[phy271115-bib-0048] Pakos‐Zebrucka, K. , Koryga, I. , Mnich, K. , Ljujic, M. , Samali, A. , & Gorman, A. M. (2016). The integrated stress response. EMBO Reports, 17(10), 1374–1395. 10.15252/embr.201642195 PMC5048378 27629041

[phy271115-bib-0049] Patel, C. V. , Handy, I. , Goldsmith, T. , & Patel, R. C. (2000). PACT, a stress‐modulated cellular activator of interferon‐induced double‐stranded RNA‐activated protein kinase, *PKR* . Journal of Biological Chemistry, 275(48), 37993–37998. 10.1074/jbc.M004762200 10988289

[phy271115-bib-0050] Pauly, M. , Angebault‐Prouteau, C. , Dridi, H. , Notarnicola, C. , Scheuermann, V. , Lacampagne, A. , Matecki, S. , & Fauconnier, J. (2017). ER stress disturbs SR/ER‐mitochondria Ca(2+) transfer: Implications in Duchenne muscular dystrophy. Biochimica et Biophysica Acta ‐ Molecular Basis of Disease, 1863(9), 2229–2239. 10.1016/j.bbadis.2017.06.009 28625916

[phy271115-bib-0051] Pedemonte, M. , Sandri, C. , Schiaffino, S. , & Minetti, C. (1999). Early decrease of IIx myosin heavy chain transcripts in Duchenne muscular dystrophy. Biochemical and Biophysical Research Communications, 255(2), 466–469. 10.1006/bbrc.1999.0213 10049732

[phy271115-bib-0052] Pennati, F. , Arrigoni, F. , LoMauro, A. , Gandossini, S. , Russo, A. , D'Angelo, M. G. , & Aliverti, A. (2020). Diaphragm involvement in Duchenne muscular dystrophy (DMD): An MRI study. Journal of Magnetic Resonance Imaging, 51(2), 461–471. 10.1002/jmri.26864 31301202

[phy271115-bib-0053] Petrof, B. J. , Stedman, H. H. , Shrager, J. B. , Eby, J. , Sweeney, H. L. , & Kelly, A. M. (1993). Adaptations in myosin heavy chain expression and contractile function in dystrophic mouse diaphragm. American Journal of Physiology, 265(3 Pt 1), C834–C841. 10.1152/ajpcell.1993.265.3.C834 8214039

[phy271115-bib-0054] Rudolph, T. E. , Mayorga, E. J. , Roths, M. , Rhoads, R. P. , Baumgard, L. H. , & Selsby, J. T. (2021). The effect of Mitoquinol (MitoQ) on heat stressed skeletal muscle from pigs, and a potential confounding effect of biological sex. Journal of Thermal Biology, 97, 102900. 10.1016/j.jtherbio.2021.102900 33863453

[phy271115-bib-0055] Selsby, J. , Pendrak, K. , Zadel, M. , Tian, Z. , Pham, J. , Carver, T. , Acosta, P. , Barton, E. , & Sweeney, H. L. (2010). Leupeptin‐based inhibitors do not improve the mdx phenotype. American Journal of Physiology. Regulatory, Integrative and Comparative Physiology, 299(5), R1192–R1201. 10.1152/ajpregu.00586.2009 PMC3774252 20844259

[phy271115-bib-0056] Selsby, J. T. (2011). Increased catalase expression improves muscle function in mdx mice. Experimental Physiology, 96(2), 194–202. 10.1113/expphysiol.2010.054379 PMC4519830 21041317

[phy271115-bib-0057] Selsby, J. T. , Ballmann, C. G. , Spaulding, H. R. , Ross, J. W. , & Quindry, J. C. (2016). Oral quercetin administration transiently protects respiratory function in dystrophin‐deficient mice. The Journal of Physiology, 594(20), 6037–6053. 10.1113/JP272057 PMC5063947 27094343

[phy271115-bib-0058] Shimazawa, M. , Ito, Y. , Inokuchi, Y. , & Hara, H. (2007). Involvement of double‐stranded RNA‐dependent protein kinase in ER stress‐induced retinal neuron damage. Investigative Ophthalmology & Visual Science, 48(8), 3729–3736. 10.1167/iovs.06-1122 17652745

[phy271115-bib-0059] Singh, M. , Fowlkes, V. , Handy, I. , Patel, C. V. , & Patel, R. C. (2009). Essential role of PACT‐mediated PKR activation in tunicamycin‐induced apoptosis. Journal of Molecular Biology, 385(2), 457–468. 10.1016/j.jmb.2008.10.068 PMC4026198 19007793

[phy271115-bib-0060] Smith, L. R. , & Barton, E. R. (2014). Collagen content does not alter the passive mechanical properties of fibrotic skeletal muscle in mdx mice. American Journal of Physiology. Cell Physiology, 306(10), C889–C898. 10.1152/ajpcell.00383.2013 PMC4024713 24598364

[phy271115-bib-0061] Spaulding, H. R. , Ballmann, C. G. , Quindry, J. C. , & Selsby, J. T. (2016). Long‐term quercetin dietary enrichment partially protects dystrophic skeletal muscle. PLoS One, 11(12), e0168293. 10.1371/journal.pone.0168293 PMC5158046 27977770

[phy271115-bib-0062] Spaulding, H. R. , Quindry, T. , Hammer, K. , Quindry, J. C. , & Selsby, J. T. (2019). Nutraceutical and pharmaceutical cocktails did not improve muscle function or reduce histological damage in D2‐mdx mice. Journal of Applied Physiology (1985), 127(4), 1058–1066. 10.1152/japplphysiol.00162.2019 PMC7276925 31295065

[phy271115-bib-0063] Spencer, M. J. , Croall, D. E. , & Tidball, J. G. (1995). Calpains are activated in necrotic fibers from mdx dystrophic mice. The Journal of Biological Chemistry, 270(18), 10909–10914. 10.1074/jbc.270.18.10909 7738032

[phy271115-bib-0064] Stedman, H. H. , Sweeney, H. L. , Shrager, J. B. , Maguire, H. C. , Panettieri, R. A. , Petrof, B. , Narusawa, M. , Leferovich, J. M. , Sladky, J. T. , & Kelly, A. M. (1991). The mdx mouse diaphragm reproduces the degenerative changes of Duchenne muscular dystrophy. Nature, 352(6335), 536–539. 10.1038/352536a0 1865908

[phy271115-bib-0065] Tronel, C. , Page, G. , Bodard, S. , Chalon, S. , & Antier, D. (2014). The specific PKR inhibitor C16 prevents apoptosis and IL‐1beta production in an acute excitotoxic rat model with a neuroinflammatory component. Neurochemistry International, 64, 73–83. 10.1016/j.neuint.2013.10.012 24211709

[phy271115-bib-0066] Valentine, R. J. , Jefferson, M. A. , Kohut, M. L. , & Eo, H. (2018). Imoxin attenuates LPS‐induced inflammation and MuRF1 expression in mouse skeletal muscle. Physiological Reports, 6(23), e13941. 10.14814/phy2.13941 PMC6286898 30548229

[phy271115-bib-0067] Xiao, J. , Tan, Y. , Li, Y. , & Luo, Y. (2016). The specific protein kinase R (PKR) inhibitor C16 protects neonatal hypoxia‐ischemia brain damages by inhibiting neuroinflammation in a neonatal rat model. Medical Science Monitor, 22, 5074–5081. 10.12659/MSM.898139 PMC5207129 28008894

[phy271115-bib-0068] Zadvornyi, T. , Lukianova, N. , Mushii, O. , Pavlova, A. , Voronina, O. , & Chekhun, V. (2023). Benign and malignant prostate neoplasms show different spatial organization of collagen. Croatian Medical Journal, 64(6), 413–420. 10.3325/cmj.2023.64.413 PMC10797232 38168522

